# A model of serial order problems in fluent, stuttered and agrammatic speech

**DOI:** 10.1016/j.humov.2007.07.004

**Published:** 2007-10

**Authors:** Peter Howell

**Affiliations:** Department of Psychology, University College London, Gower Street, London WC1E 6BT, England, United Kingdom

**Keywords:** Serial order of speech, Stuttering, Agrammatic aphasia, EXPLAN

## Abstract

Many models of speech production have attempted to explain dysfluent speech. Most models assume that the disruptions that occur when speech is dysfluent arise because the speakers make errors while planning an utterance. In this contribution, a model of the serial order of speech is described that does not make this assumption. It involves the coordination or ‘interlocking’ of linguistic planning and execution stages at the language–speech interface. The model is examined to determine whether it can distinguish two forms of dysfluent speech (stuttered and agrammatic speech) that are characterized by iteration and omission of whole words and parts of words.

## 1 Introduction

Fluent speech control occurs when the right words are spoken in their correct position. Speech is dysfluent when it deviates in either of these respects. A range of models has been developed to explain why the wrong words are sometimes produced and/or why words sometimes appear in the wrong position. Examples are Dell and O’Seaghdha’s (1991) spreading activation model, which focuses on word selection errors, Levelt’s (1983) and Kolk and Postma’s (1997) psycholinguistic models, which address word selection and word-order problems, and Howell’s (2004a) EXPLAN model which focuses on word-order problems.

Speech disorders have been used to evaluate several of these, and other, models of language production. Foygel and Dell (2000) indicate that there are usually two steps involved in modeling. Model development: (a) starts with a model of unimpaired processing, and then (b) an hypothesis how brain damage affects these processes is formulated. They illustrate this using Plaut, McClelland, Seidenberg, and Patterson’s (1996) computational model of dyslexia. These authors: (a) used a connectionist model of word naming with normal adult readers, and then (b) went on to simulate dyslexia by removal or degradation of particular connection weights or hidden units.

Certain disorders may be more revealing about word-order problems than others. Stuttering and agrammatic aphasia are characterized, respectively, by recursive use of elements in the speech sequence, and omission of whole- and part-word words that results in the words in a message not being in the intended order. Thus, these disorders would seem to be appropriate for examining word order. With this in mind, the current article outlines a model for the serial order of spontaneous speech and applies it to some of the characteristics of stuttered and agrammatic speech.

## 2 A model of the serial order of speech

The model of serial order used here is EXPLAN, where the acronym stands for execution and planning (see Howell, 2004a, for a detailed discussion). The model maintains that (a) linguistic planning and (b) motor programming and execution are independent processes, and focuses on accounting for how information is exchanged between these two modules. The critical feature for performance to be fluent is to synchronize the timing between planning and execution (at least some of the plan for the next section of speech needs to be available immediately after current motor output has been programmed and executed).

The critical performance parameter for the planning and execution modules is the time they take to complete. The model recognizes that there are several linguistic stages within planning (semantic, syntactic, lexical, morphological, phonological, phonetic and prosodic). The time needed to complete each component stage depends on the complexity of the processing required within that particular stage. Linguistic output starts to be generated at the top stage (semantic) and progresses down the hierarchy to phonetic and prosodic levels.

EXPLAN suggests how the component stages in planning are arranged. This indicates what impact the constituent stages have on generation of linguistic output. Successive stages within linguistic planning overlap, so a lexical representation commences before the syntactic form is completed, phonological representations begin to be built up for different word candidates before final lexical selection has been made, and phonetic strings for a word start to be activated once phonological information starts to accrue. Generally speaking, representations tend to build up left to right, simultaneously at all stages. Thus, the initial word in a syntactic constituent will be generated before later ones, early syllables in words before subsequent ones, the phonological representation of each syllable onset tends to build up before its nucleus and coda and, typically, this produces a phonetic string in left to right order.

There are several different segmental units within the planning process appropriate to the different stages (words, syllables, phonemes). Activation profiles represent the way the complete linguistic plan for the selected unit builds up. The units employed here are words. The first word in an utterance might be a function word. These words occur frequently and are structurally simple, e.g., the conjunction “and” and the pronoun “I”. Activation rate buildup would be rapid for these words, but less rapid for those function words which have somewhat more complex linguistic properties. For instance, all personal pronouns are single syllables with CVC structure, whereas “around” is an adverb (also a function word), is bisyllabic and ends with a consonant cluster (see Dworzynski, Howell, & Natke, 2003; Howell & Au-Yeung, 2007; Howell, Au-Yeung, Yaruss, & Eldridge, 2006, who document phonetic differences between function and content words for various languages). The rapid buildup can be represented by a sharp slope on their activation function. The point of maximum activation represents the time at which all the linguistic processes for the function word are completed. Mention has been made of parallel activation (overlap) of the linguistic representation of a word at different stages. Planning for words that occupy subsequent slots in an utterance also takes place in parallel. Planning of a second function word in the utterance starts after a delay, but before the plan of the first function word is complete, and activation increases at the same rate as with the first function word if they are equivalent in terms of complexity.

**Fig. 1:**
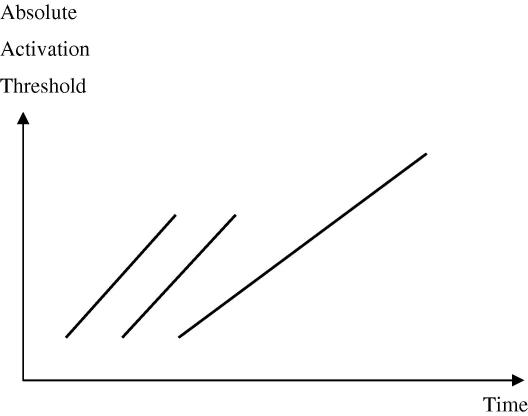
Activation buildup representing two function words and a content word.

The next word in the sequence might be a content word, e.g., the noun “strontium” or the verb “prancing” which are words that occur less frequently than function words and are phonetically more complex. The activation profile is delayed, like that of the second function word. As content words are, on the whole, linguistically more complex and longer than function words, their activations build up more gradually (lower slope on activation functions) and if they have more elements, they have to reach a higher level of activation. The buildup of activation (planning) of two function words followed by a content word, such as for the sequence “in the morning”, is shown in Fig. 1.

**Fig. 2:**
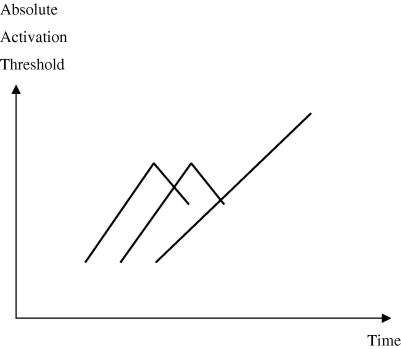
Activation buildup and decay representing two function words and a content word.

So far all that has been shown is that if you have three words whose planning starts before offset of the previous item, they will reach maximum activation in the order in which their plans commenced and be executed in sequence. EXPLAN also has an account for what happens in the execution process. Execution involves all the processes concerned with generating output, starting with the linguistic representation supplied from the planning process, and this process is reflected in the timing pattern of speech output (rather than segmental errors). Processes that are specific to output modality, such as motor programming of the articulators, occur in execution, not planning. The activation of a word that has reached its maximum will decay during the time it is executed. The activation level after it has been executed depends on how long the word takes to utter and the decay rate of activation. More complex plans persist for longer than simpler ones. This is represented as activation that decays at the same rate as it is built up. Thus if a word builds up rapidly to full activation, it will decay rapidly too. In the following figures, decay of activation is shown for the time from full activation to the end of execution time for this word (although, of course, decay of activation continues at the same rate from this point). The activation level of the word that has just been produced at the end of execution (i.e., after decay) relative to the other words whose activation is building up determines which word will be produced next in sequence. The word that has highest activation is produced. Thus, in the example in Fig. 2, function words 1 and 2 have been produced and activation has decayed, and the content word is fully activated and will be produced next in sequence.

Relative difficulty of material impacts on both error-prone processes at linguistic planning and generation of motor timing patterns at execution. The points where there is a change from easy to difficult material (from function to content words here) pose potential problems of coordination between linguistic planning and motor execution that can result in fluency failure. The problem at these points stems from the fact that the time allowed for buildup of the current segment is too short when a preceding easy segment occurs that can be executed rapidly. The particular problem arises for one or more of several reasons, including the different rates of activation and decay of easy and difficult words, as well as the output rate the speaker sets. A point to stress is that the alternation between easy and difficult words is revealing with regards to operation at the language–speech (planning–execution) interface. This does not imply that planning units at other levels are not important. For instance, syntactic units have a role in utterance planning that may impact on the timing pattern of output. It is then possible for syntactic effects to lead to different levels of difficulty which combine with other sources to determine patterns where easy/hard words alternate. A possible case of this is positioning of pauses, which seem to appear at points where easy/hard words alternate and at syntactic boundaries, which do not involve a change in difficulty of words (Ferreira, 1993). Fluent speech requires speech execution rate to be set at or below that required so that even when a difficult item follows an easy one, the later item (content word) will have reached full activation and the earlier item (function word) will have decayed to less than that level.

This section has shown that the activation profiles shown for words of different complexity produce the output words in the proper sequence if decay rate over the time needed to execute a word is included and if the rule is applied that the word that will be produced next has highest activation. Activation has to be highest, but not necessarily full, for the next word candidate to be produced. The situation where activation is highest but not at maximum represents a situation where speakers initiate speech before planning is complete (as proposed in models like that of Levelt, 1989). As outlined earlier in this section, in these cases only the early parts of the utterance being planned will be available. Problems can arise when planning is not rapid enough to keep up with execution rate. There are two main ways in which these problems can arise: when speech rate is high or when planning the next item in the sequence takes a long time. The following three sections describe how these might operate and lead to disruption in serial order, using stuttered speech as an example.

## 3 Types of fluency failure in the spontaneous speech of speakers who stutter

In our work, fluency failures are divided into two classes (called ‘stalling’ and ‘advancing’). Stallings are characterized by repetition of one or more words spoken in their entirety (usually function words) or pauses (filled and unfilled). Examples would be “in, in the morning” (single function word repeated), “in the, in the morning” (two function words repeated), and “er yesterday” (filled pause before a content word). Advancings are where a speaker produces only the first part of a word (typically a content word). Examples would be “in the mmmmorning” (prolongation of the first phoneme), “in the buh buh beginning” (part-word repetition) and “in the be/ginning” (where “/” signifies a word break, in this case between the first and second syllable).

To explain why fluency failures are divided into these types and why the class names stalling and advancing are used, it is necessary to explain the contextual unit we use for speech analysis of English. This is the phonological, or prosodic, word, abbreviated to PW (Selkirk, 1984). This unit, as applied to English, always contains a content word, and may or may not be preceded and/or followed by function words. “In the morning” is a PW of the form FFC. “He hit him” would be a PW with a final function word (i.e., of the form FCF). As noted earlier, although function and content words are referred to, the EXPLAN account applies more generally to hard- and easy-to-produce words. PW are particularly useful as their structure divides easy and hard elements in a clear-cut way. After a stretch of planned material that consists of a sequence of easy items followed by a difficult one, it is likely that the next item in the sequence will not be at full activation. Transitions from function to content words (or more generally, easy to hard items) are revealing about such transition points and, more particularly, reflect the interface between linguistic planning and motor execution. Other units (e.g., syntactic elements or spatio-temporal patterns) may be appropriate for specifying the operation within the linguistic or motor systems respectively.

Stallings occur almost exclusively on function words that precede the content word in PW. Thus a speaker might say “he [pause] hit him”, “he he hit him” but not “he hit him him” (see Au-Yeung, Vallejo Gomez, & Howell, 2003; Dworzynski, Howell, Au-Yeung, & Rommel, 2004; Howell, 2004b; Howell, Au-Yeung, & Sackin, 1999, for further discussion about PW). The role of stalling is to delay the point in time where the difficult (content) word has to be produced. This delay arises because the plan of the content word is not ready for output and ‘buys’ extra time for the remaining planning to take place (which is why this class of fluency failure is given the name ‘stalling’).

Pausing is one manifestation of stalling. Repeating one or more whole function words is another. Word repetition is allowed in EXPLAN, as the plan of a word that has just been produced can be reactivated for output, borrowing an assumption made by Blackmer and Mitton (1991). While the reactivation is happening, planning of the next word can take place and this continues during the time the repeated word is being executed.

Advancing represents the case where fluency failure again arises because the plan of the content word is not ready (see the earlier comments about incomplete plans), but in this case, the speaker carries on, runs out of plan, cannot complete the word and prolongs, repeats or breaks the word with a hesitation at the point where the plan ceases. The same problem lies behind stalling and advancing: the plan for the content word is not ready in time. Details of these processes can be revealed by analysis of dysfluencies in PWs. The following two sections examine whether stalling and advancing can be simulated using the assumptions outlined above.

## 4 Simulation of stalling

Two key concepts in EXPLAN are: (1) The next word that will be produced in the sequence is the one with highest activation (although the activation is not necessarily complete); (2) The plans of previously-produced words can be reactivated from the point where they have decayed and are then re-executed. Selecting words with highest activation can result in the next word produced not being the next one in the planned temporal sequence (in contrast with the situation described in Section 2). In this section it is shown how these two properties can result in stalling. Stalling can occur after the initial function word in a PW has been produced and some of its activation has decayed. Nevertheless, the activation level of this function word can still be high and may exceed that of the subsequent content word being planned whose activation is still building up but has not reached its maximum. This situation arises because the speaker is producing speech at too rapid a rate prior to the content word, so its plan is not ready in time. The speaker may deal with the situation where the content word plan is not ready by increasing the time to execute the initial function word to obtain more time to complete planning of the content word. Stalling, as described above, is a way of slowing execution rate and should occur when execution rate is high, leading to the content word plan not being ready in time.

**Fig. 3:**
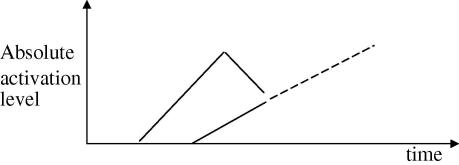
Activation and decay parameters for the situation leading to stalling for a PW consisting of a function word preceding a content word. The solid lines represent the activation states for the function word (left) and content word (right) at the point where the speaker has just finished uttering the function word. The dotted lines indicate the activation for the content word still has some way to go before it reaches full activation. Stalling results in this case because the function word has decayed but activation is still greater than the activation for the following content word and, consequently, the function word is repeated.

The case of single function word repetition is shown in Fig. 3 for the situation where execution rate is too high for production of the PW “he stood” (FC). The function word has been uttered once. Note that at this point, activation for the content word is lower than that of the function word. Note also that this is so despite the fact that some decay of activation of the function word has occurred.

## 5 Simulation of advancing

In this section, it is shown how the content word that follows one or more function words in the planned sequence, can have highest, but not full activation. As the content word has the highest activation level, it will be produced next in order, but as its activation is not full, only the first part of its plan will be available. This situation can lead to the different forms of advancing (prolongations, part-word repetition, word breaks).

**Fig. 4:**
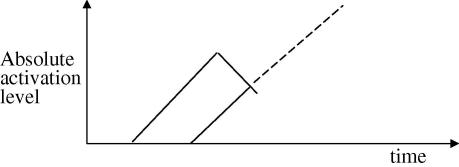
Activation and decay parameters for the situation leading to advancing for a PW consisting of a function word preceding a content word. The solid lines represent the activation states for the function (left) and content word (right) at the point where the speaker has just finished uttering the function word. The dotted lines indicate the activation for the content word still has some way to go before it reaches full activation. Advancing results in this case because the function word has decayed below the activation level of the following content word and, consequently, the content word is initiated even though it still has some way to go before it reaches full activation (indicated by the dotted line).

If execution rate is matched to the complexity of the constituents in an utterance, the PW will be spoken fluently, as discussed in Section 2. If activation rate (reflecting planning) of the content word is slowed slightly or execution time is decreased (speech rate is high), the function word that has just been produced can have a higher activation than the content word that is next in the planned sequence. This leads to function word repetition, as discussed in the previous section. If activation rate is slowed further or execution time decreased, because of the rapid decay on function words, the situation arises where the content word has higher activation than the function word although activation is not full (as shown in Fig. 4).

If speakers initiate execution of the content word based on its partial plan (advancing), dysfluency involving the first parts of the word ensues. One might ask how the different forms of advancing (e.g., prolongations, part-word repetitions, etc.) arise when the plan for the content word is incomplete. The ideas about stalling that were used in earlier parts of the text, when applied to part of a plan, would lead to prolongation and part-word repetition. Prolongation would arise when the onset consonant alone is available (which happens mainly on fricatives, laterals and nasals) particularly in cases where the consonants are continuants (Howell, Hamilton, & Kyriacopoulos, 1986). Part-word repetitions arise when the plan is complete up to the onset-nucleus boundary. This type of fluency failure tends to occur mainly on interrupted consonants (Howell et al., 1986). Word breaks occur when the plan is complete to the point between onset plus nucleus, but typically not beyond that point in the syllable (i.e., not to the coda). Speakers would break out of the loop when the subsequent part of the plan is completed.

Stuttered speech has been used as one form of disordered speech for the illustrations so far in this article. In a more generalized approach, an adequate model of serial order problems would have to apply to other disordered forms of speech as well (particularly those involving problems of repeated or omitted words). A particular form of speech that has differential effects on function and content words is agrammatic aphasic speech. Speakers with this condition are often described as having telegraphic speech. The main features are loss of function words and loss of inflectional endings on verbs and nouns (Tissot, Mounin, & Lhermitte, 1973). The way these features are simulated in EXPLAN is described in the following section.

## 6 Agrammatic aphasic speech (telegraphic speech)

The hypothesis concerning how damage leads to agrammatic speech is that activation buildup (and the constituent psycholinguistic processes behind it) is not affected by brain damage per se, but the result of that process (as appears in Figs. 1–3) is masked by neural noise caused by the damage sustained. Neural noise is conceived as global impairment to brain function in general (here all the processes in planning and execution). So, for instance, in Fig. 2 this noise might be represented as a horizontal line lying above the maximum function word activations (all these words are masked and none can be uttered) but below the maximum activation the content word reaches (the bottom part is masked, but the peaks of activation poke above the noise floor, so they are not completely masked and the words can be uttered). This proposal is radically different to Plaut et al.’s (1996) notion that CNS damage in the case of dyslexia affects the connection weights or hidden units which would change the activation function itself. Ingestion of substances like mercury and alcohol (Hunt, 1993) and certain physical injuries (Alm, 2005) lead to diffuse, rather than focal, central nervous system processing problems such as those described as neural noise. While a process like that Plaut et al. (1996) described is appropriate for focal lesions to areas which have a specific functional role in processing, problems that arise from injury that affect more diffuse parts of the brain (affecting linguistic planning and motor execution) need to be modeled differently.

The modeling assumptions receive circumstantial support from the fact that the features of agrammatic aphasia (loss of function words and inflectional endings on content words) both occur in individuals with Down syndrome. Chapman (1995) and Fowler (1995) reported that these speakers drop function words. Eadie, Fey, Douglas, and Parsons (2002) compared Down syndrome, children with specific language impairment and controls. Problems were reported for both the Down syndrome and specific language impairment children for word-final inflections similar to those that have been reported in agrammatic aphasics. The similarity between agrammatic and DS speech has not been commented on previously. This is, perhaps, not surprising as one is a genetic problem that affects early childhood, whereas the other arises typically in adults through neurological trauma. It seems unlikely that Down syndrome and agrammatic aphasia result in a lesion to the same area of the brain, making models like those of Plaut et al. (1996) unlikely candidates for a common model of both disorders. On the other hand, both disorders might result in high levels of neural noise that affect planning and execution processes. The remainder of this section examines whether assuming that planning and execution processes are masked by neural noise could explain how the features common to agrammatic speech and Down syndrome speech arise.

To examine this hypothesis about disruption to speech sequences after brain damage, function words were examined in more detail. Up to now, these words have been treated as a homogeneous class, though it was noted in Section 2 that their activation would vary with their linguistic complexity. In some studies on agrammatic speech, function words have been broken down into different grammatical types and were ordered in terms of the difficulty they pose. The order Caramazza and Berndt (1985) give for omission is presented in the left-hand column of Table 1. (Note these authors do not differentiate types of pronouns.) The right hand column gives order of difficulty of these word classes for people who stutter calculated from Brown (1937). Brown started with 23 “parts of speech” from function and content word classes. These were ordered with respect to the percentage of stuttering each produced, in a study on 32 adults who stuttered. Brown collapsed the 23 categories into what he called the eight conventional parts of speech and his collapsed categories and rankings are used directly for articles, prepositions and conjunctions. The pronoun category in Table 1 includes only personal and possessive forms (not relative forms like “who” and “what”). Brown collapsed auxiliaries (which are function words) with other verbs (which are content words). The auxiliaries were treated as a separate class in Table 1 (i.e., the rank given for this class in his list of 23 categories was used).

There is a high correlation between the two columns with only prepositions and auxiliaries in a different order and then only by one ordinal position. Thus, agrammatic aphasics omit the words that speakers who stutter find easiest. This is what would be expected on the basis that there is some gradation in activation of function words. In particular, the easiest function words (top of column 2) have lowest levels of activation, which makes them most susceptible to masking, and the hardest function words have the highest level of activation which increases their chance of having activation that is above the noise background. EXPLAN accounts for function word loss or retention in terms of whether activation is masked or not.

It was noted earlier that inflectional endings on verbs and nouns are affected in agrammatic speech. For English, two classes of function words carry information about content word inflections: pronouns and auxiliaries. Pronouns carry information about plurals on nouns and verbs whilst auxiliaries carry information about past tense and participles on main verbs. Plural marking on nouns and verbs, and the present participle on verbs (which defines aspect, the temporal flow of events) have both been reported as likely to be retained (Tissot et al., 1973). Past tense, on the other hand, is more likely to be lost (Tissot et al., 1973).

The hypothesis to be examined is whether the likelihood of loss of function words relates in turn to which inflectional endings on content words are likely to be lost. For this analysis, it is assumed (a) that the appropriate unit for understanding the loss/retention of inflectional endings is the PW, and (b) that the function words most likely to be retained are those at the bottom of column one, Table 1.

Plural inflections on nouns and verbs are carried by personal and possessive pronouns, which usually precede the noun in PW. (Personal and possessive pronouns were examined separately from relative pronouns for the stuttering data in Table 1 because the latter do not signal plural inflection.) All pronouns occur second from the bottom in the list of function words lost in agrammatic aphasics (second easiest for these speakers). If personal and possessive pronouns are retained, this would, in turn, allow the plural on a noun or verb to be retained (when appropriate). This situation holds statistically, although it is possible to generate exceptions where the pronoun in a PW does not signal plural (e.g., “My buddies and I were shot”).

When a present participle is created on a main verb (e.g., going from “I read” to “I am reading”), an auxiliary is inserted. Auxiliaries are not the easiest function words for agrammatic aphasics (second from the top, so second most difficult), but neither are they the hardest. On the occasions they are retained they may signal a present participle ending on the following main verb. Once again this is not likely to be absolute, as when a phrase is interposed between the auxiliary and main verb.

In both the former cases, function word retention could potentially explain why inflectional endings are retained and this would be consistent with EXPLAN. It is harder to account for loss of past tense information. Past tense is not carried by prepositions, articles, pronouns, or conjunctions but it is by auxiliaries. A speaker would have to lose auxiliary information to lose past tense. However, this is the opposite of what was argued in connection with the present participle.

This section closes with some general remarks concerning how the EXPLAN account relates to, and differs from, other proposals about agrammatic speech. The closest explanations to the current one in the aphasia literature are the family of ‘processing accounts’ (e.g., the mapping hypothesis by Linebarger, Schwarz, & Saffran (1983) and Kolk’s (1995) desynchronization account). These accounts attempt to explain agrammatism as a usage, rather than a representation/planning, phenomenon. There are also ways in which the EXPLAN account could be modified so that it could be linked more directly to representational views. The properties of function and content words employed in the EXPLAN account (that function words tend to be easy and carry information about inflectional endings on content words) need not be inherent to word class per se, but may reflect the type of processing needed to be done on different words and whether that processing needs to extend across words. The account then becomes more similar to the way in which a linguist would look at these phenomena. A linguist would point out that auxiliaries are functional projections, while content words are terminal nodes in syntactic trees. The hierarchical breakdown of functional categories hypothesis of agrammatic production models the phenomena discussed along these linguistic lines (Friedmann & Grodzinsky, 1997; Hagiwara, 1995). There is one study on the placement of adverbs in the speech of an agrammatic bilingual that seems to support the view that processing demanded by words, rather than word class, affects retention/loss of words in agrammatism (Alexiadou & Stavrakaki, 2006). These authors performed a constituent ordering task with an English-Greek agrammatic aphasic patient. The patient had problems in adverb ordering in English but not in Greek, which suggests that word class is not responsible for the difference. Adverbs in Greek have a rich morphology that uses particles and verb inflections, whereas English relies more on modal and auxiliary verbs to mark distinctions. Thus, English relies more on words in the adjacent context. If contextual information was lost in this patient, this would explain why adverbs in English, but not in Greek, are affected.

Verb regularity might also be thought to constitute a problem, insofar as the account applies only to regular verbs. The process outlined would not operate on irregular verbs. Thus, this particular proposal requires that regular and irregular verbs are processed either by different mechanisms or for there to be dissociable impairments to a single processing system. There is evidence that has been interpreted for both such possibilities. Acquired damage to the left perisylvian cortex, for instance, leads to impaired regular past tense processing, leaving irregular past tense processing intact (Tyler et al., 2002). Damage to the middle and inferior temporal lobe leads to impaired irregular past tense processing, leaving regular past tense processing intact (Marslen-Wilson & Tyler, 1997). Both the dual and the single mechanism accounts of language processing acknowledge this double dissociation. The difference is in how the double dissociation is explained: According to the dual mechanism account (Longworth, Keenan, Barker, Marslen-Wilson, & Tyler, 2005; Marslen-Wilson & Tyler, 2003; Ullman, 2001), two separate mechanisms underlie the processing of regular and irregular forms; according to the single mechanism account, the double dissociation is due to phonological (for regulars) or semantic (for irregulars) impairments in a single unified processing system (Bird, Lambon Ralph, Seidenberg, McClelland, & Patterson, 2003; Joannise & Seidenberg, 1999; McClelland & Patterson, 2002a, 2002b). Two predictions that would follow from the EXPLAN account are: (1) that irregular inflections should be retained in patients who lose function words and inflectional endings; and (2) function words and inflectional endings would be retained in those patients who lose irregular inflections.

EXPLAN assumes that the production and perception systems in the brain act independently. This is consistent with the classic assumption about agrammatic aphasics who exhibit impaired production with unimpaired comprehension. There is evidence for differential performance in comprehension and production by agrammatics (see for instance, Kim & Thompson, 2000, for a recent demonstration of unimpaired comprehension of verbs/nouns but impaired naming in agrammatism). It has, however, also been convincingly demonstrated that agrammatic comprehension is impaired to some extent too (Shapiro, Zurif, Carey, & Grossman, 1989; Zurif, 1980).

## 7 Conclusions

The EXPLAN account of fluent speech, stuttering and agrammatism consider that these behaviors arise out of the way the linguistic planning and motor execution processes interface. In the case of the two disordered forms of speech, the problems arise when difficult units are being planned for the next position in a sequence whilst simple to execute units are produced in the prior sequence. The account emphasizes the crucial role of alternating easy/hard material at the interface. It does not deny that problems can arise at either the planning or execution stages, it simply emphasizes the importance of the way the interface operates. Insofar as problems specifically at the planning or execution stages have not been included, the account is partial (as, indeed, are accounts that claim that one of these stages alone is responsible for fluency problems).

It has been shown how EXPLAN generates stallings and advancings. Important concepts have been drawn from Blackmer and Mitton’s (1991) work that introduced precompiled filler words, Levelt’s (1989) notion of partial planning, the PW segmental unit drawn from Selkirk’s (1984) studies as well as previous work by the team at UCL on the way different phonetic classes lead to problems at different points in syllables (Howell et al., 1986). It has been shown that the account explains some features of stuttered and agrammatic speech.

## Figures and Tables

**Table 1:** The left-hand column gives the order of omission of function words in agrammatic aphasic speakers extracted from Caramazza and Berndt (1985), the center column gives order of difficulty of function words for stutterers derived from Brown (1937) as described in the text and the right-hand column gives the activation levels at each extreme

Caramazza and Berndt (1985)	From Brown (1937)	Activation
**Hard (lost first**)	**Easy (least stuttering)**	**Low**
Articles	Articles	
Auxiliaries	Prepositions	
Prepositions	Auxiliaries	
Pronouns	Pronouns	
Conjunctions	Conjunctions	
**Easy**	**Hard**	**High**
